# Negotiating participation in conversations about care planning between people living with mild neurocognitive disorders and their healthcare proxies: a single case analysis

**DOI:** 10.1093/geront/gnag011

**Published:** 2026-02-15

**Authors:** Anca-Cristina Sterie, Francesca Bosisio, Eve Rubli Truchard, Ralf J Jox, Laura Jones

**Affiliations:** Chair of Geriatric Palliative Care, Palliative and Supportive Care Service, Lausanne University Hospital and University of Lausanne, Lausanne, Switzerland; Service of palliative and supportive care, Lausanne University Hospital and University of Lausanne, Lausanne, Switzerland; School of Management and Engineering of Canton Vaud (HEIG-VD), University of Applied Sciences and Arts Western Switzerland (HES-SO), Yverdon-les-Bains, Switzerland; Chair of Geriatric Palliative Care, Palliative and Supportive Care Service, Lausanne University Hospital and University of Lausanne, Lausanne, Switzerland; Service of Geriatric Medicine and Geriatric Rehabilitation, Lausanne University Hospital and University of Lausanne, Lausanne, Switzerland; Chair of Geriatric Palliative Care, Palliative and Supportive Care Service, Lausanne University Hospital and University of Lausanne, Lausanne, Switzerland; Chair of Geriatric Palliative Care, Palliative and Supportive Care Service, Lausanne University Hospital and University of Lausanne, Lausanne, Switzerland; Service of palliative and supportive care, Lausanne University Hospital and University of Lausanne, Lausanne, Switzerland

**Keywords:** Dementia, Interaction, Advance care planning, Autonomy

## Abstract

**Background and Objectives:**

The time between diagnosis with a mild neurocognitive disorder (MND) and definitive loss of decisional capacity presents a window of opportunity to participate in advance care planning (ACP). To implement early planning, we need to know how to promote healthy relationships between people with MND and their healthcare proxies. Our objective is to examine how people with MND and their proxies discuss engagement in ACP and how proxies orient toward the ability and right of the person with MND to provide answers.

**Research Design and Methods:**

We undertake a conversation analysis of an interview related to ACP between a researcher, a person with MND and their proxy, recorded in Switzerland.

**Results:**

The way in which the proxy and the researcher orient to the person with MND changes throughout the interview. In the beginning, they recognize the person with MND as a knowledgeable and competent participant (facilitating answers or allowing her to speak first). Later, practices bypass the speakership primacy of the person with MND (correcting or describing her as dependent). The person with MND sometimes resists these stances, for example, by contradicting her proxy’s answer.

**Discussion and Implications:**

Our study contributes to understandings of how the epistemic rights of a person with MND to participate and provide information within interactions are constructed variably. This has implications for promoting the people with MND’s individual and relational autonomy in interactions and decision-making and developing awareness-raising resources about how to improve the conditions of decisional autonomy of people with MND.

Neurocognitive disorders involve a decline in cognitive functioning (McDonald, 2017) that gradually reduces a person’s ability to make complex decisions (Darby & Dickerson, 2017). The time between diagnosis of mild neurocognitive disorder (MND) and eventual loss of decisional capacity presents a window of opportunity to participate in getting one’s affairs in order, including involvement in treatment decisions and advance care planning (ACP) (Bosisio et al., 2018, 2021; Tetrault et al., 2022). In the beginning of the MND trajectory, there may be cognitive deficits in understanding and retaining given information, while the capacity to appreciate one’s own illness and express choices are preserved (Hamann et al., 2011), which means that people can still have meaningful interactions and inputs. Furthermore, the presence of healthcare proxies can be a resource for people with MND to navigate the intricacies of decision-making. In order to implement early planning for people with MNDs, we need to know how best to lever the information given to them but also to protect the relationships with their healthcare proxies—during and beyond—decisional processes.

Linguistic-inspired methodologies, such as a conversation analysis, are particularly adapted to examining how MND affects the interactional skills needed for participation in everyday discussions and care-related acts. Prior research has shown how people with dementia deal with difficulties talking about recent events (Jones, 2015), finding words (Lindholm, 2008), or using pronouns without a clear reference (Hamilton, 1989). Increasing pragmatic difficulties are also observed, related to interactional competency: with advancing neurocognitive disorders, people have more difficulty decoding and responding to open-ended questions (Allwood et al., 2017; Dooley & Barnes, 2022; Walker et al., 2023) and ultimately only minimally contribute to the interaction (Hydén et al., 2022; Majlesi et al., 2022; Schneider et al., 2019).

The predictable trajectory of dementia in relation to loss of medical decision-making capacity in the (relatively near) future often changes the dynamic in relationships and, therefore, interactions between people with MND and their healthcare proxies. Thus, there is a joint impact as innate loss of ability is reinforced by lowered expectations from family and caretakers and reduced opportunities to engage in day-to-day living activities, leading to further deterioration of the ability to participate in such activities. Relationship quality, even for a person with MND, is lower between partners where one spouse has neurocognitive disorders (Clare et al., 2012).

Previous research between adults with intellectual disabilities and their healthcare proxies has reported that companions “intervene” in patient responses to varying degrees (Antaki & Chinn, 2019; Kindell et al., 2017). For example, Landmark et al. (2021) have identified ways in which companions correct people with dementia via correcting statements, inviting them to self-correct and disagree. Lindholm (2015) has specifically investigated companion’s reactions to people with dementia’s confabulations (unknowingly uttering false information) and reported several techniques which are used to either accept, remain noncommittal, or correct the person. Literature also highlights that people with dementia may accept or resist being corrected and re-establish their authority over these domains (Black, 2011; Landmark et al., 2021). While similar tendencies are reported in medical interactions involving patients retaining full neurocognitive capacity and their healthcare proxies (Pino & Land, 2022), some aspects seem to be exacerbated by the diagnosis of dementia.

Nevertheless, information is scarce on how MND impacts interactions, particularly during discussions about future medical decisions and about how healthcare professionals, proxies, and people with MND might negotiate their participation to the interaction and the decision-making in real time.

## Objective

The objective of this research was to examine the ways in which people newly diagnosed with MND and their healthcare proxy (hereafter, proxy) discuss their past, current, and future engagement in ACP, in an interview with a researcher. We identified all moments throughout the data in which both the proxy and person with MND intervene to respond to the same question (posed by the researcher) and provide information about an aspect that primarily concerns the person with MND. We focused on how participants orient towards the ability and right of the person with MND to provide answers and negotiate entitlement to do so.

## Methods

### Data

Data for this analysis come from a pilot interventional study of ACP for people with MND (2017–2019), that was approved by the local ethics committee (number: 2018-00785). A total of 23 semi-structured interviews were conducted by ACS and FB, in French, pre- and post-ACP intervention, with people with MND, who retained decision making capacity according to the Montreal Cognitive Assessment (Nasreddine et al., 2005), and their proxy(s). The objective was to gain insight into the reasons for requesting or accepting to engage in ACP, prior experiences of similar interventions (e.g., writing advance directives or discussing wishes with someone else) and their experience of the intervention. Screening was conducted by FB, ERT, and RJJ. These interviews were audio-recorded and transcribed verbatim by a professional transcriber. Thematic analyses were conducted to describe the acceptability and feasibility of the intervention, as published elsewhere (Bosisio et al., 2021). Here, we present a secondary analysis of a phenomena which were identified inductively during primary analyses.

### Analysis

Secondary analysis of the data involved recurrent data sessions between ACS, LJ, and FB, and focused on practices that seemed specific to interaction with people with MND. This initially led to an investigation of instances in which proxies speak in the name of the person with MND, published elsewhere (Sterie et al., 2023). For the present analysis, two authors (ACS and LJ) read all interviews (*n* = 20) in parallel to identify as phenomena of interest instances in which participants negotiate the entitlement of the person with MND to provide information about an aspect concerning themselves. Then, each extract was individually discussed during a data session among the two authors to reach analytic agreement. Practices pertaining to the negotiation of the entitlement of the person with MND were identified and inventoried. This includes, for example, cases when the proxy answers a question in the name of the person with MND, clarifies or corrects the latter, when the researcher orients questions about something concerning the person with MND towards the proxy, but also episodes in which the person with MND challenges or resists these impositions by producing overlapping talk, taking over the conversation, or contradicting the proxy. Each type of practice encountered in the collection was exemplified with corresponding examples from the dataset.

Data were analyzed with conversation analysis. Conversation analysis resides in a finely grained analysis of recorded conversations and focusses on how participants interact in order to accomplish ordinary as well as interactionally challenging tasks, such as starting a conversation, introducing and closing down a topic, formulating a request, managing a disagreement, and so on (Sacks et al., 1974). The analysis describes the processes whereby participants accomplish, coordinate, and interpret their reciprocal actions. Two literature reviews highlight the recent surge in conversation analytic based studies on communication with people who have communication and cognitive difficulties and display its relevancy as methodology on this topic (Dooley et al., 2015; Kindell et al., 2017).

We build, in particular, on the concepts of epistemic primacy and access. Conversation analytic studies have demonstrated that speakers in an interaction build their turns at talk with a particular attention to knowledge (Heritage, 2012a, 2012b). The way in which, for example, questions, assessments, or responses are designed can often display, among other things, epistemic status (the person’s rights, responsibilities, and obligations to know something) and stance (how these roles are enacted in the moment). This dimension seems particularly interesting in interaction with people living with MND, as memory problems are one of the first signs of the syndrome. As such, an array of conversation analytic studies have focussed on the mobilization of knowledge and forgetting within this population (Black, 2011; Landmark et al., 2021; Nilsson, 2022; Schrauf, 2020; Svennevig & Landmark, 2019).

We present our results based on the analysis of a single case of an interview collected during the aforementioned project. This interview was chosen because it is particularly rich, as it exemplifies several interactional practices and resources through which the researcher, the proxy, and the person with MND negotiate the latter’s entitlement to participate to the interview and provide informative answers. The extracts that we selected seemed particularly clear in themselves from an analytically point of view and at the same time illustrative of the variation of these types of practices. These practices were also identified in other extracts of the broad dataset, however, this interview contained the most examples. This case-based method was selected because it offers a dynamic and real-time view into the progression of these practices along the course of the interview, thus affording a reflection about how roles and relationships between participants highlight the interactional challenges brought on by the MND diagnostic.

### The conversation

The interview participants are the researcher (a medical sociologist), the person with MND (female, 78 years old), and her daughter (59 years old), who is equally her healthcare proxy and a practicing nurse. All participants are female, which is why we will refer to them as such throughout the text. The interview took place early in 2019, at the home of the person with MND: All three participants are sitting at a round table. The person with MND and her daughter have already agreed to participate in an ACP intervention, which was programmed for a later date.

Names were replaced by pseudonyms in the extracts. The extracts present one part of a conversation (from 12:48 a.m. to 03:23 a.m.) that lasts for 28:50 minutes overall, that we have separated into six parts, according to the topics that are pursued. The precise timing at which the extract starts during the interview is presented in the title, in brackets. In these extracts, there is a researcher who is the interviewer, a healthcare proxy, and the person with MND. To facilitate the reading and also endorse a person-centered language, we refer to the participants according to their pseudonyms: Ivy (person with dementia), Ana (healthcare proxy, who is Ivy’s daughter), and Mia (researcher). Transcription conventions according to the Jeffersonian system (Jefferson, 2004) are available in Table 1.

**Table 1:** Transcription conventions.

Convention	Signification
**=**	No discernable break between the turns/latching conversation
**[**	Point of overlap onset
**]**	Point of overlap end
**(3.4)**	Length of silence, measured in seconds and tenths of seconds
**(.)**	Micro pause (less than 0.2 of a second)
**:**	Lengthening or stretching of the sound
**–**	Cut-off or self-interruption
.	Falling intonation
,	Continuing intonation
**?**	Talk ending with rising intonation
**h**	Hearable outbreath
**·h**	Hearable in breath
**underline**	Emphasized talk
**°no no°**	Talk is quieter than surrounding talk
**>no no<**	Talk is faster than surrounding talk
**((word))**	Transcriber’s description
**£yes£**	Word is uttered with smiling voice

## Findings

Here, we present six extracts which unfold sequentially, and we identify the practices through which the researcher, the healthcare proxy (hereafter, proxy), and the person with MND negotiate the latter’s entitlement to participate to the interaction and provide informative answers. We start with a stepwise analysis to show how the “infringement” of the person’s epistemic claims, both from the researcher and from the proxy’s side, becomes increasingly acute as the conversation unfolds, as well as how the person with MND challenges these stances. Several resources are employed, such as question and response design, reference to memory, descriptions depicting the person with MND as dependent. For an overall comprehension of the phenomena, we synthesize the specific practices we identified in this conversation at the end of the findings section with links to the particular extracts in which they are encountered.

### Exploring prior discussions of advance directives

Extract 1 is from the very beginning of the conversation. Here, we see the interviewer designing questions to be answerable by a person with potential memory problems; the proxy steps in to answer a question originally directed at the person with MND when the latter admits not being able to answer.

The interviewer produces a request for information directed to Ivy. Lacking visual data, this orientation only becomes clear in lines 5 and 7 as, in the absence of a response, the interviewer produces candidate answers that designates Ivy (“your daughter,” “your daughters”). From the start, the accomplishment of the request is contingent on Ivy’s memory (“do you remember”, line 1). This opens the possibility of a lack of response and its presumed justification, as the interviewer topicalizes “memory” from the very start of the interview. However, while allowing Ivy to demonstrate competency, this design also highlights their incompetence (Mikesell, 2009).

Following two attempts to obtain confirmation to candidate answers (line 5 and 7), Ivy states that she does not remember (line 9). This is produced in soft voice while, in overlap, the interviewer launches another effort to retrieve an answer. The switch from an open information request to a request making relevant the validation of a specific information shows that the latter is considered, by the interviewer, to be more manageable by Ivy, as it requires less cognitive and interactional effort. An avenue of participation is kept open for Ivy and adapted to her lack of response which typically indicates difficulty.

After another marked silence (line 11), the proxy self-selects to answer the question, first validating interviewer’s candidate answer (“yes”, line 12) then providing details. After Ivy states not being able to answer and when the interviewer is still waiting for an answer, the proxy displays entitlement to intervene and provide the information requested. While the proxy answers in Ivy’s place, she does so in her own name, since the answer is indexed to her (“my,” “me”). As such, it is misaligned with the interviewer’s question and, in doing so, it changes the parameters of the interaction, as the proxy assumes equal access to knowledge and thus speakership rights.

Ivy provides minimal acknowledgement, and the interviewer accepts the information as valid and continues the conversation.

### Exploring instigators for prior advance directives

Extract 2 follows on directly from Extract 1, as the interviewer continues her questions. The researcher explicitly invites both Ivy and her proxy to respond, which they do at the same time.

**Extract 1:**
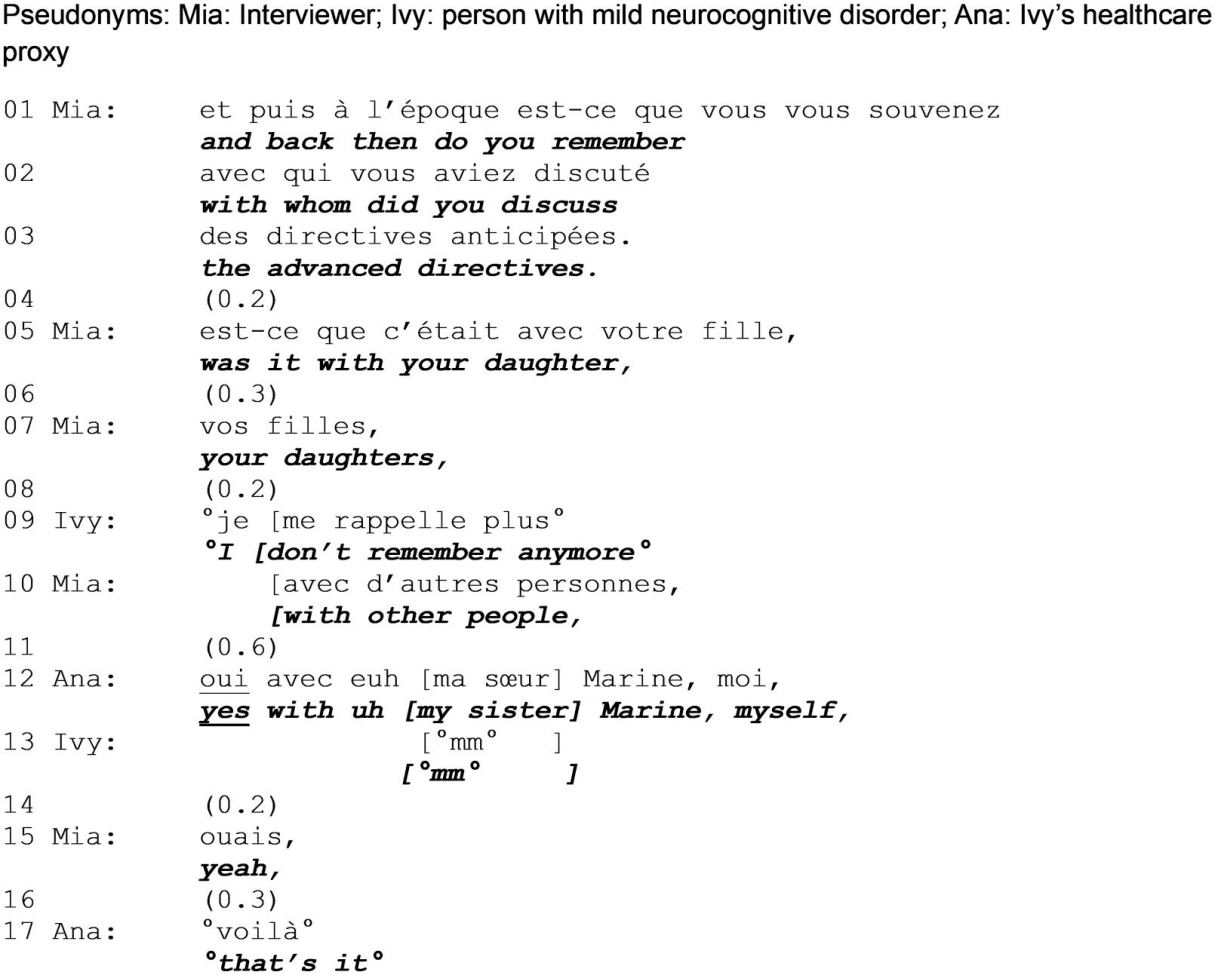
Prior discussion of advance directives (start: min 00:48).

**Extract 2:**
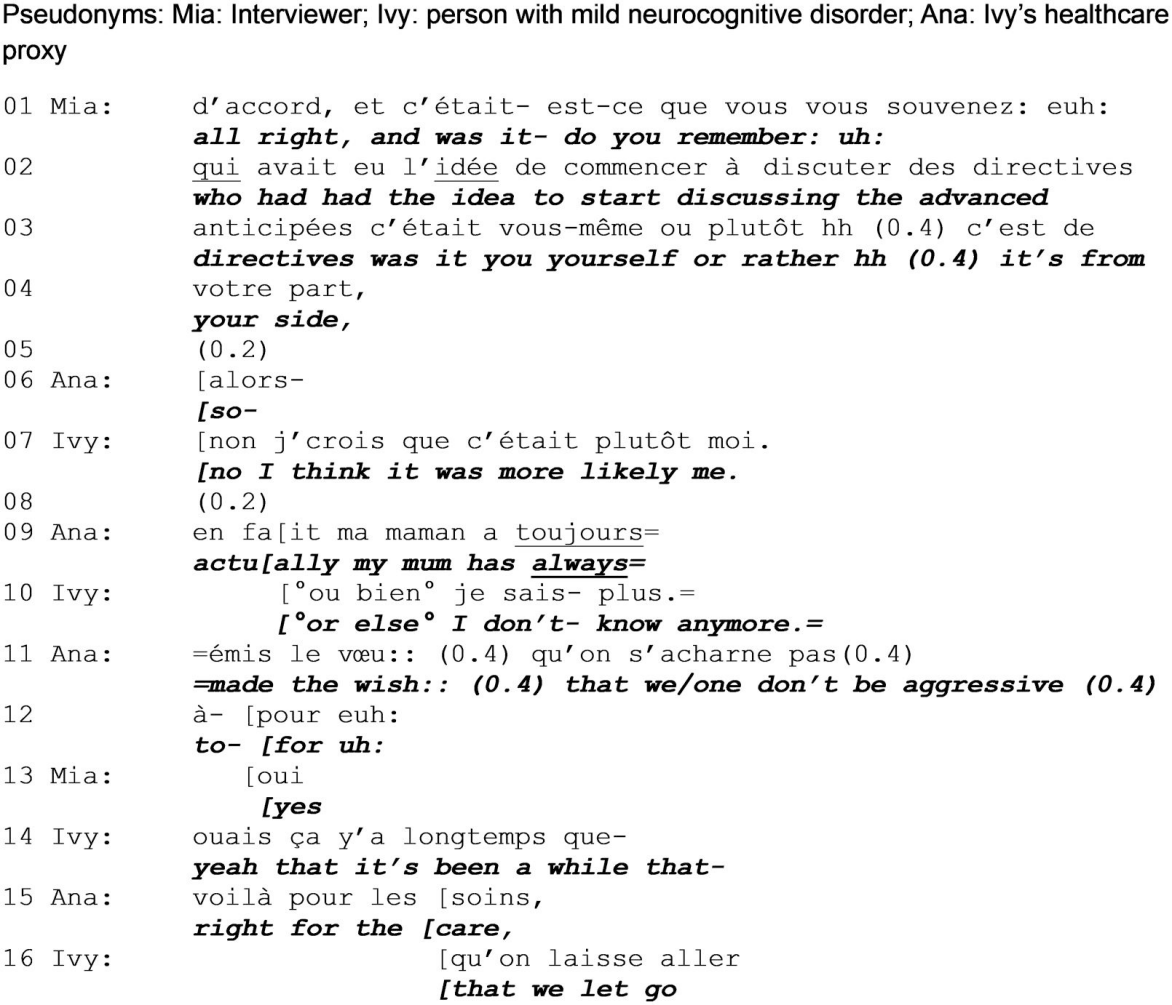
Instigators for prior advance directives (start: min 01:03).

As in Extract 1, the interviewer launches a request for information, again indexing memory as a potential challenge and barrier to obtaining a response. The request is fitted, while it is being produced, to the contingencies of the interaction: the fact that Ivy was unable to respond to the previous question but that it nevertheless concerns an aspect over which Ivy has epistemic primacy over the proxy, as it is designed to address both participants (“it was from you yourself or rather from your part”).

The proxy self-selects to answer, starting to do so (line 6) at the same time as Ivy (line 7). The proxy self-interrupts to let Ivy produce the answer, thus showing that Ivy holds the epistemic primacy (to know and to answer) in this situation. Ivy’s initial “no” contradicts the interviewer’s last candidate answer but also the proxy’s self-selection to respond. The response is produced with an epistemic stance marker (“I think”), that emphasizes the speaker’s personal perspective. While she also semantically downgrades the certainty of her answer (“more likely me”), Ivy reiterates her entitlement over the knowledge and her right to express it.

The proxy initiates what is prefaced with what might seem to be an alternative, challenging answer (“actually,” line 9), thus claiming equal rights to contribute to the response. In her answer, she refers to Ivy as a third party (“my mother”), thus not only speaking for her but also about her (Nilsson et al., 2018). As she speaks, Ivy also initiates talk in overlap (line 10), claiming unknowing (“anymore”) and thus discounting the accuracy of the information that she contributes. This shows that Ivy understands the proxy’s initiation as projecting a contestation of her answer. The proxy produces a clarification of Ivy’s response, giving more context and content about the activity. This goes beyond the interviewer’s question but is relevant in this context. The proxy establishes that Ivy did initiate the decision but not necessarily the discussion. Her response is also more fitted to the type of content the researcher seeks, by providing more information. However, the proxy’s intervention also involves Ivy. As the proxy reports that Ivy “always” (stated with emphasis) made her wishes known to not have aggressive care, she also aligns with Ivy’s statement that they initiated the discussion (even if they did not initiate the ACP discussion referenced by the interviewer here). This could account for the proxy providing this additional information here; it could be a way to answer the question that also aligns with Ivy’s prior answer and fulfils the information needs of the researcher.

### Exploring reasons for engaging in ACP

Extract 3 is from further along in the conversation. In it, the proxy starts answering a question oriented toward Ivy; she interrupts to let Ivy answer and then provides additional information.

**Extract 3:**
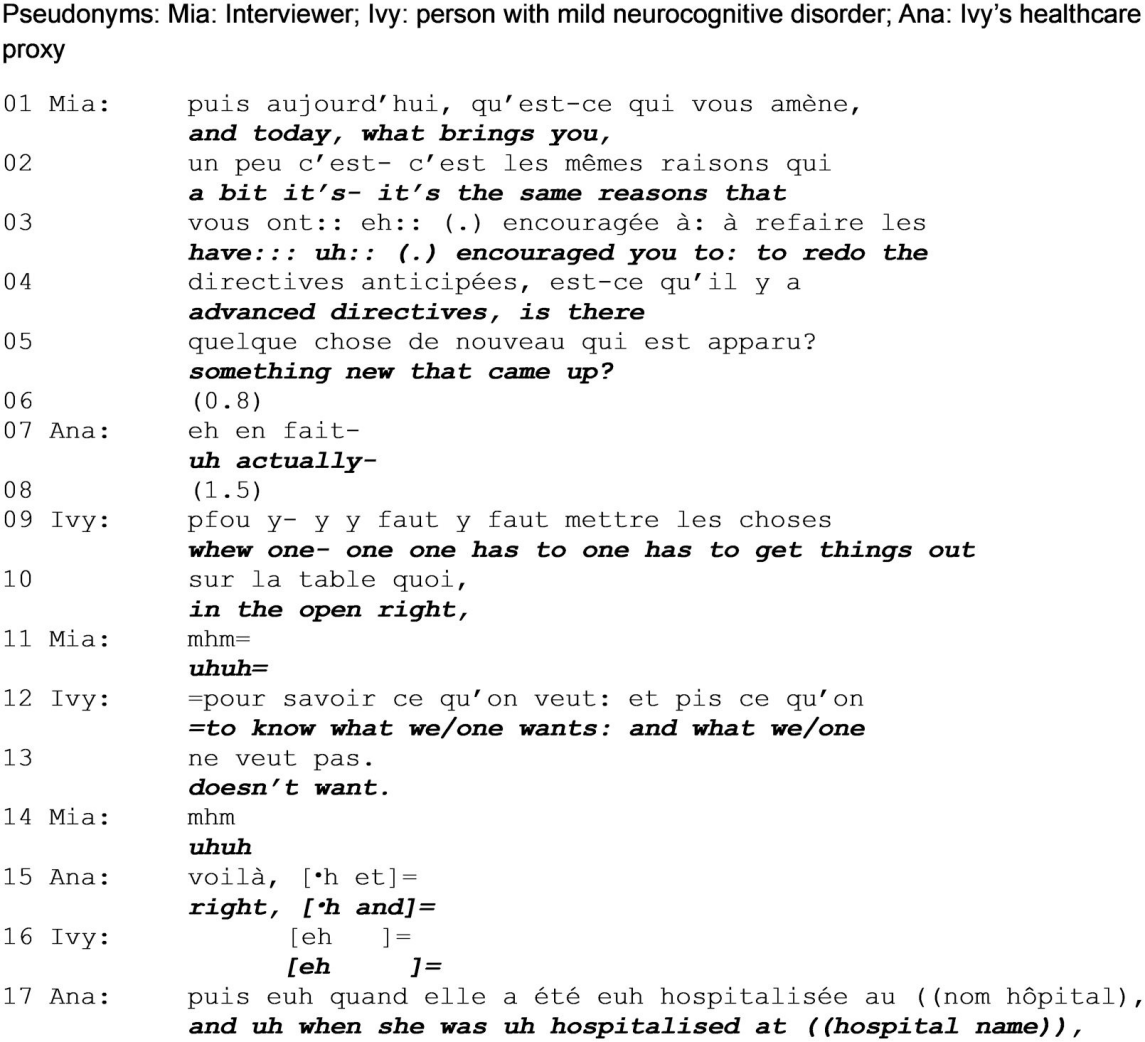
Reasons for engaging in ACP (start: min. 01:56).

The interviewer’s next request for information follows the previously established request pattern of an open elicitation (line 1, “what brings you”) followed by candidate answers (lines 2–5). After a significant silence, the proxy self-selects to answer (line 7), her turn-initial signaling the start of a more complicated explanation (“actually”). However, she self-interrupts, thus creating an opportunity for Ivy to answer.

In her response, Ivy delivers an idiomatic expression (lines 9–10 and 12–13) that is ambiguous, referring to the decision as “the things” and self-referring as a generic entity (“what one wants and what one doesn’t want”). This is also a formulaic expression, a string of words that appears stereotypical, prefabricated (Wray, 2024), and is often employed in certain cultures and contexts. Multiple studies report that people with language disorders, including dementia, produce idioms particularly when facing word-finding difficulties, as they are automatically triggered and thus more easily deployed that other structures such as storytelling (Wray, 2024). Literature has also shown that idiomatic expressions are often encountered in the context of a complainable (Drew & Holt, 1988). In this particular case, Ivy might be answering with what is presented as being a benign, un-complicated reason to a question that seeks more depth (as suggested by the turn-initial “pfou”). Ivy might also anchor her answer in the fact that idioms are also hard to challenge (Kitzinger, 2000); in this context, in which Ivy’s status is repeatedly challenged, it might serve to better position her authority and entitlement.

As in Extract 2, the proxy uses Ivy’s answer as a pivot and self-selects to clarify it (line 15). After validating the information Ivy provided (“right,” line 15), she introduces a follow-up (“and then when,” lines 15 and 17) which actually provides very different (though not contrasting) information. While Ivy seemingly focused on the reason for doing advance directives, the proxy focusses on the specific context in which the family opted into the research.

In Extracts 4–6, the interviewer discusses Ivy’s current daily planning. In Extract 4, Ivy and her proxy provide conflicting answers to the researcher’s question.

**Extract 4:**
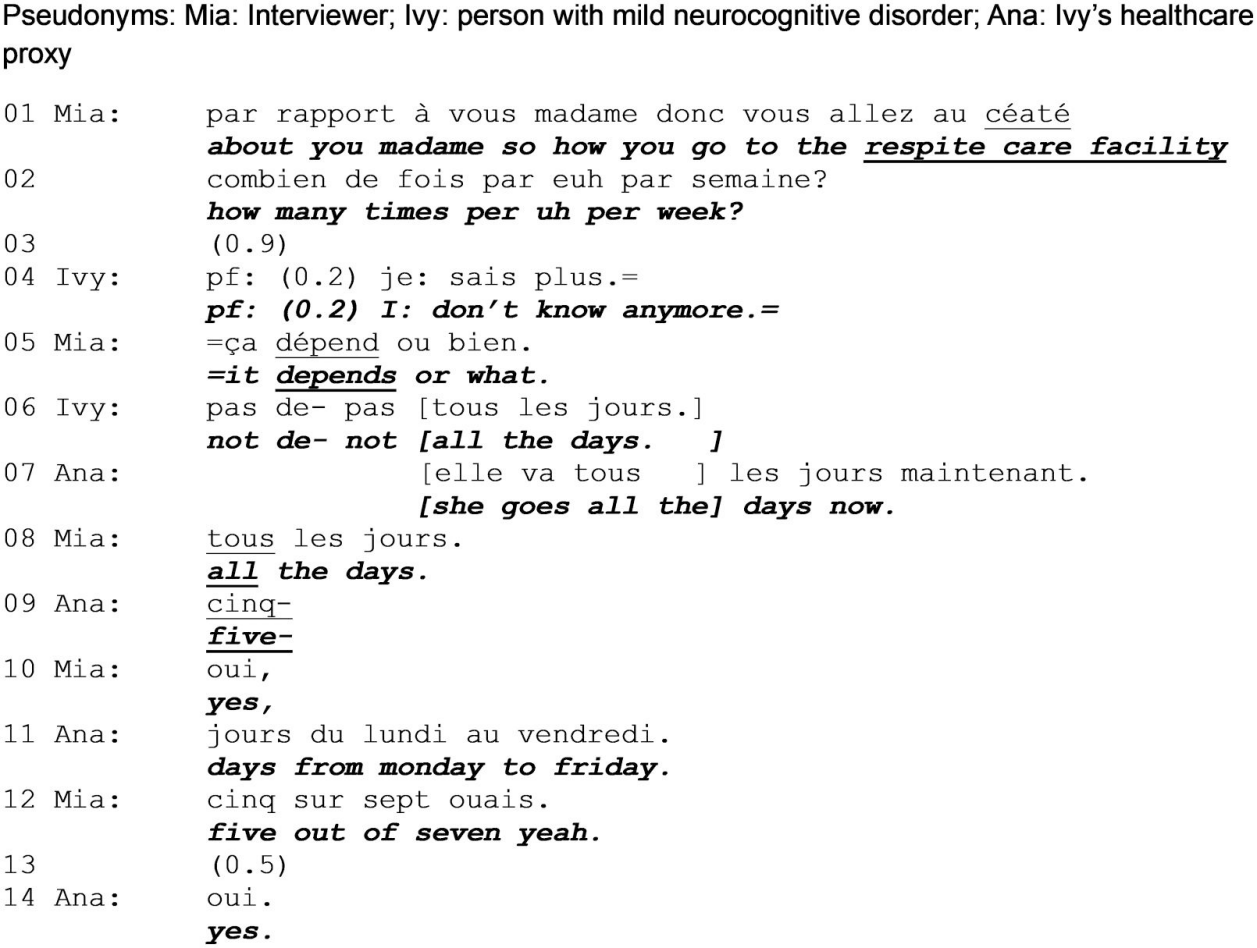
Attendance at respite care nursing home (start: 02:45).

### Exploring attendance at respite care nursing home

In Extract 4, the interviewer’s request for information is indexed as directly addressing Ivy (“about you madame”), containing no mitigators accounting for memory, thus displaying consideration that Ivy is both competent and authorized to provide this information. After Ivy avows lack of memory (line 4), the interviewer continues with a candidate answer (line 5), which doesn’t contain an informative response but a potential reason for not remembering or for a difficulty in retrieving information (i.e., because it varies). Ivy formulates an answer coherent with this theme (line 6), that is vague yet acceptable. In overlap, the proxy corrects Ivy, using the same formulation (“all the days”), revealing her prior answer as a confabulation (Lindholm, 2015). In this way, the correction is anchored in Ivy’s own words, which makes it even more powerful and contradictory of Ivy’s response. The exchange continues without Ivy’s intervention.

### Exploring organization of professional home help

In Extract 5, the researcher orients the question toward the proxy; Ivy and the proxy again produce conflicting responses.

**Extract 5:**
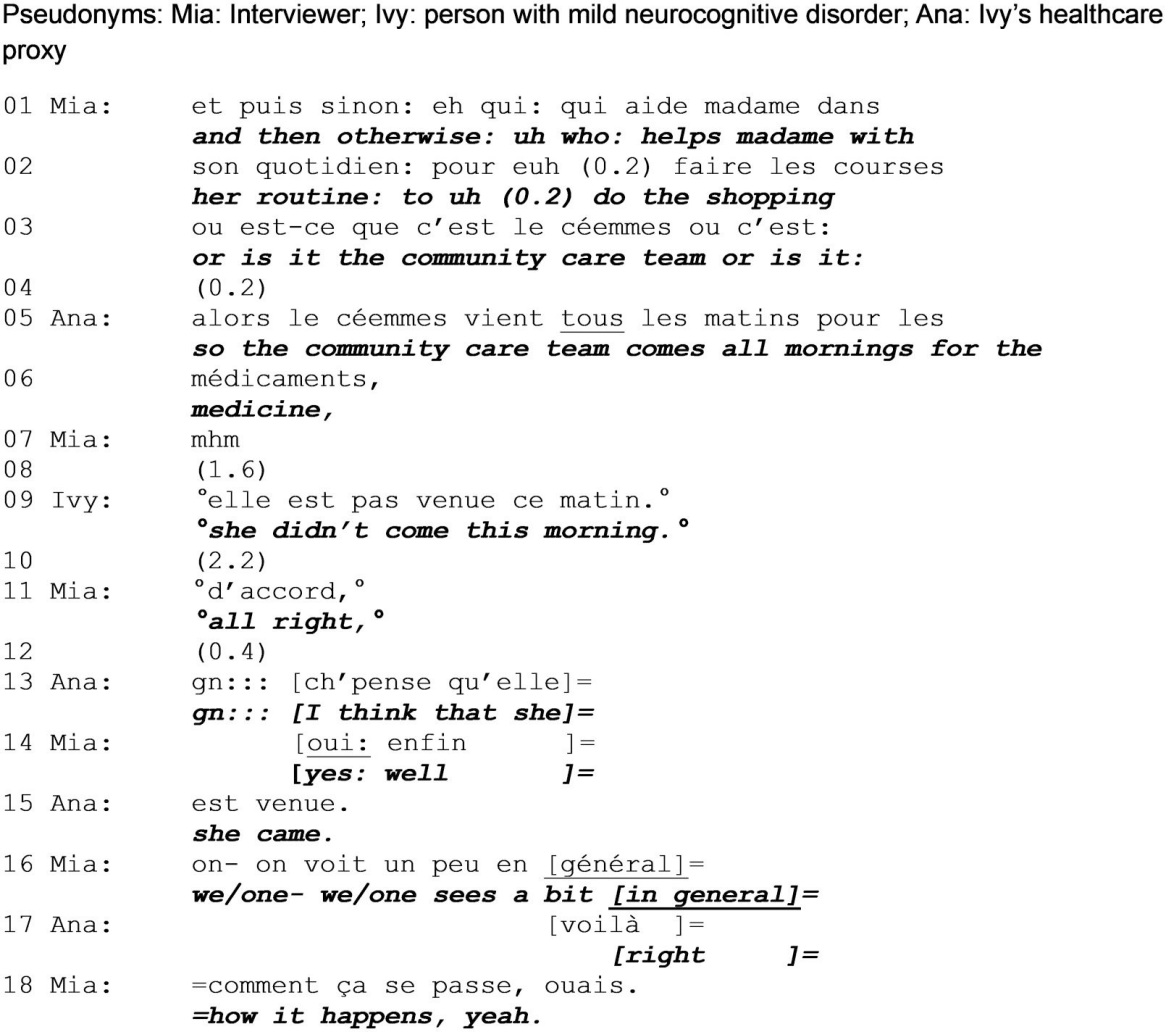
Organization of professional home help (start: 03:01).

The interviewer’s next question still focusses on Ivy but addresses only the proxy (“who helps madame,” line 1), in the same format employed previously (open elicitation followed by candidate answer), rendering speaking on behalf of Ivy interactionally necessary. Previous research shows that once a particular dyadic question-response pattern has been established with the proxy of a person who has memory difficulties, further interaction is unlikely to include the person with memory difficulties (Karnieli-Miller et al., 2012). The proxy responds (lines 5 and 6) and her continuous intonation projects that there is more to come. Yet, she does not continue immediately and after a significant pause (line 8, 1.6 s) Ivy steps in to produce information that is contrary to the proxy’s (line 9). The remark is made with soft voice, displaying low entitlement to speak. Through this remark, Ivy resists the interviewer’s and proxy’s stance in relation to who is entitled to speak about the issue at hand, to the point at which she contests this entitlement. After a considerable silence (line 10, more than 2 s), the interviewer takes up the remark at its face value (line 11) while the proxy contests it (lines 13–14). The proxy’s turn-initial (“gnnn,” line 13) shows that she is dealing with an interactionally problematic situation, as indeed Ivy does have first-hand access to the information and can only be contradicted on the basis of cognitive problems. The interviewer acknowledges that the subject has been discussed in enough depth and mitigates the detail required for the question (lines 14 and 16), with which the proxy agrees (line 17), thus de-escalating the interactional problem. In sum, Extracts 4 and 5 show Ivy giving an answer that is displayed as being incorrect by the proxy.

### Exploring organization of non-institutional help

In Extract 6, Ivy protests against the way in which the proxy describes her as dependent of social support to maintain a safe and healthy day-to-day life at home.

**Extract 6:**
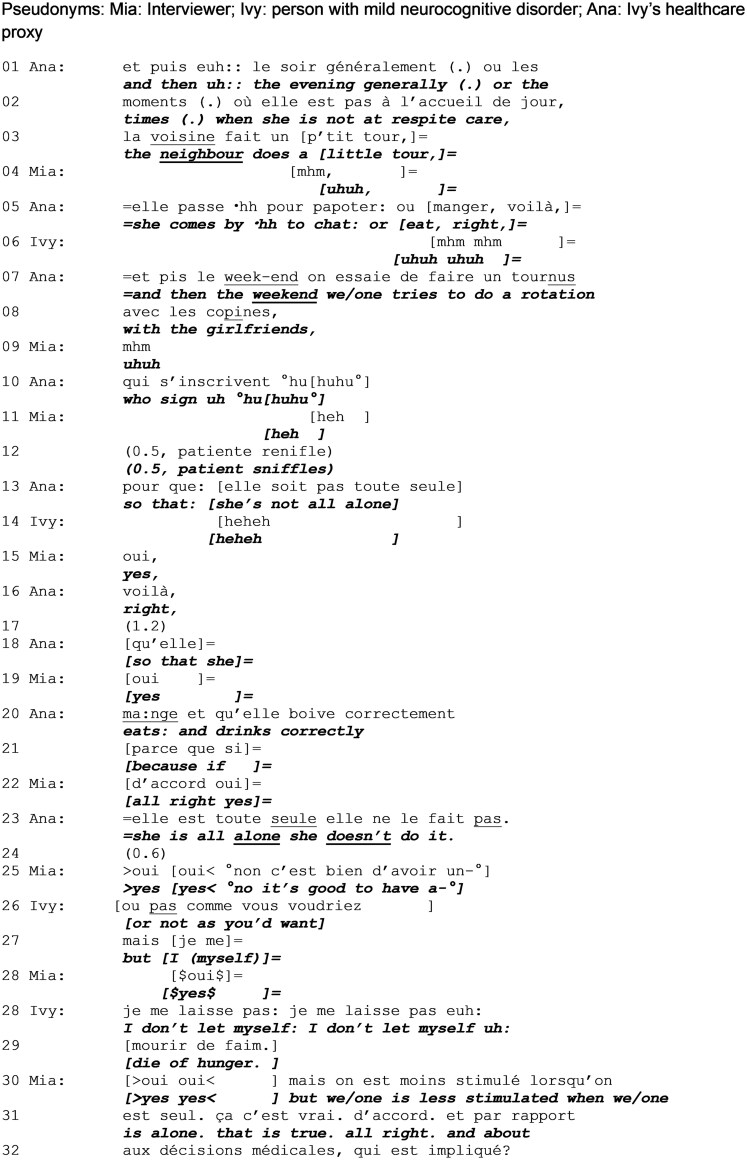
Organization of non-professional help (start: 03:33).

The conversation continues with the proxy talking about Ivy’s daily organization. In lines 1 to 13, she offers a very detailed and structured description of a plan that is organized in temporal slots (“evening” vs. “weekend”) and according to intervening people (“the neighbour,” “her friends”). Ivy corroborates this account (line 6).

Until line 11 the proxy’s account focuses on tasks, however, starting with line 13 she focuses more on reasons for the entourage’s intervention and on Ivy herself, depicting her mother as dependent, initially socially (“not to be alone,” line 13), and then in terms of health habits (“eat and drink correctly,” line 20), finishing off with a straightforward assessment about her (in)capability to perform activities of daily living (line 23). Ivy laughs at the details of needing to be provided with companionship (line 14), but contests the proxy’s final remark (line 26). In doing so, Ivy introduces the idea of alternative interpretations of what is happening, and claims still being able to deal with the most basic needs (“I don’t let myself die of hunger,” lines 28–29). Here, the formulaic response is used as a complaint (Drew & Holt, 1988). Given its prospective character, it is also difficult to oppose. The interviewer tries to pacify both parties by finding a more diplomatic way of explaining the need for companionship, and guides the conversation toward another subject. The conversation continues without the proxy providing a response to the Ivy’s contradiction, which can be heard as a strong form of disagreement.

Throughout her turn (lines 1–23), the proxy employs a technical vocabulary (“rotation”) that denotes her profession (a nurse) but, together with the intricate details (“making a list”) displays the need to actively manage Ivy’s life and works up Ivy as incapable of living without this elaborate support system. Furthermore, the laughter particles (line 10) display that she is aware of making remarks that are potentially face threatening for her mother. The description finishes off with the mother’s contestation, that while explicitly addressing only the very last remark about her incapacity to drink and eat by herself, can be heard as a general misalignment in regard to the image that the proxy is building up.

## Inventory of practices

In Table 2, we provide an inventory of practices and resources through which the interviewer and the proxy manage the role of the person with MND in this conversation and display a certain orientation to it, by recognizing/acknowledging or challenging her entitlement to participate. We describe them in terms of how the practices display the speaker’s orientation toward the epistemic access of the person with MND (first-hand access to the information), right (being entitled to speak about something), competence (being “able” to participate independently of the fact of being entitled to), and participation (how the practice offers more or less opportunities for the person with MND to participate or excludes the person with MND from the conversation). In Table 3, we provide an inventory of practices and resources through which the person with MND claims back their epistemic rights. The tables synthesize findings from this particular interview, though the practices inventoried are also encountered in several other conversations in our dataset.

**Table 2:** Interviewer and proxy practices that orient toward the epistemic access and competence of the person with MND pseudonyms: Ivy: person with mild neurocognitive disorder; Mia: Interviewer; Ana: Ivy’s healthcare proxy.

Practices	Example	Description
**Requests for information**		
**Addressed to Ivy (marked)** **Addressed to Ivy but indexing memory** **Addressed only to Ivy**	“About you madame, so you…” (Ex. 4, line 1)	The request is addressed to Ivy and indexes her name or personal title. It considers Ivy as the only one having the epistemic primacy to the information, recognizes her right to utter this information in interaction, and considers her as capable of doing so (especially as it’s an open question, so Ivy is considered capable of retrieving content).
	“Do you remember…” (Ex. 1, line 1)	The request is addressed to Ivy and recognizes their epistemic primacy to the information, and right to utter this information in interaction. It also includes a clause about memory as gatekeeper and reason for lacking competence to take up this role. Therefore, it offers a more diplomatic “way out” of answering the question but assumes the reason behind a potential lack of answer.
	“Who helps madame…” (Ex. 5, line 1)	The request concerns an issue to which Ivy has first-hand access but is addressed to the proxy. It considers the proxy as having epistemic right over a domain concerning the information and experience of Ivy. By referring to Ivy in the third person, it excludes her from the discussion (she is a subject of discussion, not a participant).
**Gives candidate answers**		
**When Ivy doesn’t answer right away** **When Ivy admits not remembering**	“With your daughter, with your daughters…” (Ex. 1, lines 5–7) “It’s from your side…” (Ex. 2, lines 3–4)	The candidate answer is formulated when Ivy, as recipient of the request, doesn’t deliver a response at the first opportunity. The candidate answer transforms the open question into a validation request. It considers Ivy as having the epistemic primacy to the information and as being entitled to respond but assumes that she might not maintain the competence to answer an open request.
	“With other people (Ex. 1, line 10) “It depends or what…” (Ex. 4, line 5)	The candidate answer is formulated when Ivy, as recipient of the request, delivers a non-answer response by which she claims memory problems. The candidate answer transforms the open question into a validation request. It considers Ivy as having the epistemic primacy to the information and as being entitled to respond but assumes that she might not maintain the competence to answer an open request.
**Answers in Ivy’s place**		
**When Ivy admits not remembering anymore** **Bypassing Ivy** **Clarifies and modifies Ivy’s response** **Continues Ivy’s response**	“Yes my sister Marine” (Ex. 1, line 12)	Proxy self-selects to answer a request directed to Ivy when Ivy delivers a non-answer response by which she claims memory problems. Proxy respects person’s right to answer by allowing time for it but jumps in when Ivy claims not being able to do so. She automatically assumes next-speakership rights. Responses are designed to show her equal epistemic access to the information and the right to answer.
	“So- ” (Ex. 2, line 6) “Actually” (Ex. 3, line 7)	Proxy self-selects to answer a request directed at Ivy when Ivy doesn’t deliver a response at the first opportunity. Proxy only respects person’s right to answer in a limited way and assumes first speakership rights. Proxy self-interrupts when Ivy starts to answer, thereby recognizing Ivy’s epistemic primacy and entitlement to speak only when Ivy claims this.
	“Actually my mum…” (Ex. 2, line 9)	Talk is subsequent to Ivy’s response. It projects a clarification as something that departs from what Ivy has produced. It claims that Ivy’s and proxy’s epistemic access to the information are equal, recognizes Ivy’s entitlement to deliver that information in interaction, but claims that the proxy has higher competence to do so in interaction.
	“Right and then…” (Ex. 3, line 15)	Talk is incremental to Ivy’s response. It confirms Ivy’s response but projects a continuation. It claims that Ivy’s and proxy’s epistemic access to the information are equal, recognizes Ivy’s entitlement to deliver that information in interaction, but claims that the proxy has higher competence to do so in interaction.
**Contradicts Ivy**	“She goes all the days now” (Ex. 4, line 7) “I think that she came” (Ex. 5, lines 13 and 15)	Proxy contradicts Ivy by stating the same formulation that Ivy delivered in the prior turn to deliver contradictory information. The contradiction is not indexed as one. It recognizes the patient’s epistemic access but implies that Ivy doesn’t maintain the competence to mobilize that information in interaction. This implicitly challenges the fact that entitlement to speak should be grounded in epistemic primacy alone. The proxy assumes equal epistemic access to the information and higher entitlement to deliver that information than Ivy.
**Works up Ivy as dependent**	“If she is alone she doesn’t do it” (Ex. 6, lines 21 and 23)	By describing the elaborate support system that is put in place for Ivy, the proxy works her up as incapable of living without help.
**Talks about Ivy as third party when the information concerns her**	“Actually my mum has always made the wish…” (Ex. 2, lines 9 and 11) “She goes all the days now” (Ex. 4, line 7) “Who helps madame…” (Ex 5, line 1)	Proxy and interviewer refer to Ivy as third party though she is participating to the conversation and the information concern her directly. No resources for achieving joint speakership are made available. Ivy becomes a subject of the talk and excluded from participation.

**Table 3:** Practices of the person with MND to claim back epistemic primacy and rights to talk pseudonyms: Ivy: person with mild neurocognitive disorder; Mia: interviewer; Ana: Ivy’s healthcare proxy.

Practices	Example	Description
**Produces overlapping talk**	“Mia: Do you remember (…) was it you yourself or rather it’s from your side Ana:[so- Ivy:[no I think it was more likely me. ” (Ex. 2, lines 1–7)	When the proxy starts to answer a question addressed to Ivy, the latter initiates talk overlapping with the proxy, which results in the proxy self-interrupting. Ivy thus claims back speakership rights as well as demonstrates herself as apt to provide an answer (linguistic capacity) as well as having epistemic access to the information seeked and being entitled to provide the answer first.
**Participates to the elaboration of a response**	“Ana: she made the wish that we don’t be aggressive to for uh Ivy: Yes that it’s been a while that- Ana: right for the care, Ivy: That we let go” (Ex. 2, lines 11–16)	When the proxy answers a question concerning Ivy, the latter initiates talk which overlaps and contributes to the proxy’s recounting of an event by confirming it and providing new, corroborating, information. Ivy asserts equal speakership rights but does so from a complementary, collaborative position.
**Formulaic expressions**	“Whew one- one one has to one has to get things out in the open…” (Ex. 3, lines 9–10)	Ivy answers a question through a formulaic expression. Such formulations have been shown to provide people with memory difficulties with resources that give the appearance of maintaining a command on grammar and language, provided they are used coherently with the context.
**Contradicting the proxy answering in her name**	“She didn’t come this morning” (Ex. 5, lines 5–9) “Or not as you’d wish but I don’t let myself I don’t let myself uh die of hunger” (Ex 6, lines 28–29)	Ivy initiates talk that contradicts the proxy’s response, about an event to which Ivy has first-hand access. She claims equal epistemic access with the proxy and claims a right to expose her side of things. In doing so, she implicitly challenges the proxy’s right to respond the question in the first place and the proxy’s stance in regards to how she describes the event.

## Discussion

Based on the example of a single interview between a researcher, a person with MND and their proxy, we examined practices and resources through which participants negotiate the entitlement of the person with MND to provide information about an aspect which concerns them. Through certain practices, the proxy and the researcher orient toward the entitlement of the person with MND to give information and thereby recognize her important role in the conversation as a knowledgeable and competent participant. These include: the researcher designing information-seeking questions as oriented toward the person with MND or providing candidate answers that facilitate the production of an answer, the proxy allowing the person with MND to speak first or interrupting the turn at talk when the latter starts to speak. Other practices bypass the speakership primacy of the person with MND and challenge her entitlement to participate to the conversation. These include: the researcher designing information-seeking questions as oriented toward the proxy, the proxy answering in the name of the person with MND without allowing her opportunities to do so first, the proxy denying or correcting the information that the person with MND shared or describing the latter as dependent. Some practices employ a combination of these. For example, certain types of clarifications produced by the proxy after the person with MND has already given an answer or continuations of their answer, while being produced as confirmations, also display a stance whereby the proxy assumes the right to contribute to the information. We also identified several practices through which the person with MND resists these stances and claims back participation entitlement, for example, by producing overlapping talk when the proxy has already started to give an answer, by elaborating or even contradicting her proxy’s answer. In addition, we documented formulaic responses that allow the person with MND to take the floor and assume speakership without, however, significantly contributing significant information to the conversation.

The sequential approach that we undertake offers a dynamic view into how these practices shift gradually, from the beginning to the end of the interview. The conversation starts off with interviewer and proxy being relatively inclusive in regard to the person with MND, creating opportunities for her participation in the interaction, but increasingly excluding her. In this sense, it reflects not only the impact of cognitive disorders on the interaction but how these are brought into the interaction and made available by the participants themselves, resulting in the person with MND being progressively excluded over time.

The right to talk about something is generally grounded in epistemic primacy (having first-hand access to that information or event). However, there are some exceptions in which we stray from this interactional rule, for example, in cases when the person with epistemic primacy doesn’t have the competence to mobilize or deploy that information in interaction. In these instances, the “person to ask” is someone else. In this sense, interaction competence is considered as more important than epistemic primacy; this is the case for children (Cahill, 2010; Stivers, 2001), migrants who don’t speak the language (Bolden, 2012), and people with dementia (Hydén, 2008; Sterie et al., 2023). This is based on predefined categories of people for whom this competence is problematic, but often, also, based on relationality, such as when a spouse is speaking on another’s behalf. When companions intervene in a discussion to clarify what their spouse says, this can be considered as an indicator of the fact that they claim “more direct knowledge of the [person’s] circumstances” (Antaki & Chinn, 2019). However, it is equally a claim of having more competency of expressing that knowledge toward a third party. In instances like we have presented here, where there are multiple dynamics at play, it is important to interpret the results in light of the near impossibility of disentangling the relational interactions between a person with MND and her daughter, who is, at the same time, a health professional and healthcare proxy, with whom a relationship has been built up over a lifetime.

Globally, our findings highlight how the proxy is faced with contrastive demands: tending to the progressivity of the interaction (the interviewer’s question have to be answered in a timely manner), but also to the main activity (providing answers that are correct and complete) and to her relationship with the person with MND (giving her time to answer, acknowledging her as participant). The delicate balance of encroaching on the accounts and experience of the person with MND and maintaining focus on the goals of the medical encounter which we see the proxy/daughter attending to her, have also been noted in Antaki and Chinn’s (2019) analyses of adults living with intellectual disabilities and their companions. Handling Ivy’s problematic conversational competence is heightened by the fact that the interaction is under observation by the interviewer. As Hydén et al. (2013) remark, “other aspects than the actual content of the conversation are important for the participants–for instance being together, supporting the positive identities both presented in the story and embodied in the socially rewarding activity that they manage to engage in, implying that the participants create and sustain a common ground.” The extracts that we present here show how the proxy/daughter draws on the closeness of her relationship with her mother to display her entitlement to sometimes ‘take over’ the reigns of the discussion. This is made accessible by referring to things that Ivy has “always” wished for (Extract 2) but also by offering detailed information about how Ivy’s care is organized, information that Ivy herself does not contribute or not in full: confirming that Ivy goes to the respite care facility 5 days per week (Extract 4), that the community care team did come in the morning of the interview (Extract 5) and organizing a “rotation with the girlfriends” (Extract 6). This information is often offered in contradiction with Ivy or by cutting her off. On the other side, Ivy advances her own agenda and understanding of habits related to her aging (Pickard, 2024; Sabatini et al., 2024), and certain limits that she does not cross (such as letting herself “die of hunger”). It is difficult, in the interpretation of the data, to disentangle to what point interactions reflect the effects of early-stage dementia and to what point they simply display typical relationship habits. This, in itself, is an important remark, as if it is difficult for researchers, it is equally difficult for other third parties who might become involved, such as health professionals or proxies.

Finally, our findings also contribute to a more general reflection about how third parties who are unfamiliar with the person with MND and their proxy (such as an interviewer) might shape interaction and contribute to including or progressively excluding the person with MND from the conversation. As Nilsson (2022) argues, these patterns can be representative of how interactions are shaped in other instances in which an unfamiliar third-party is involved, such as medical or other institutional interactions, and findings be all the more useful.

## Limitations

A single case analysis has allowed an in-depth exploration of the numerous practices in which the proxy and researcher either promote or hinder the person with MND’s participation in the discussion. However, this method does limit the generalizability of the results as they are specific to the individual context: a person newly diagnosed with dementia, participating in a meta-discussion about their intentions to participate in ACP, in a health system whereby the model is new and it is facilitated by specifically trained health professionals. In addition, while allowing an in-depth analysis, this method does not present a wider variety of conversational practices.

## Conclusion

This study focuses on people who maintain medical decision-making capacity but who have been diagnosed with MND and will therefore likely lose this capacity with the progression of the disease. Our findings show how several practices are displayed throughout the course of a longer conversation, and how they become progressively exclusionary, and how the person with MND goes from initially rather passive stance to challenging the progressive exclusion. The findings shed light on the ways in which this entitlement is worked up in interactions with people with MND, whether these are an enduring feature of social relationships or momentary expressions.

## Implications for policy and/or practice

The observations reported and discussed in this article have important implication for the autonomy of people with MND. Through a relational perspective of autonomy (Jaworska, 1999), relatives and healthcare providers caring for people with dementia are called upon to become actively involved to enable patients’ capacity for autonomy by taking the values and preferences that these people express seriously. The communication impairments people with MND experience and the epistemic rights claimed by proxies that care for them might, however, result in a paternalistic attitude, hence reducing the actual autonomy of people with MND. In this sense, our study contributes to understandings of how autonomy can be worked up within interactions between people with MND, proxies, and third parties. It provides valuable information upon which to base information sessions and awareness raising for health care proxies and relatives of people with MND, including training them to use supportive communication strategies (Folder et al., 2024), for example, with group information sessions for proxies and relatives of people with MND which include meta-reflection techniques in the form of Conversation Analytic Role Play method (Stokoe, 2014) or Conversation Analysis Based Simulation (Pilnick et al., 2023), among others. While there is an increasing number of such resources organized for health professionals, they are less systematically used and evaluated with healthcare proxies (Kindell et al., 2017). Improving how proxies and people with MND communicate will also support the establishment of concordant ACP documents that will improve care at a later point when the dementia diagnostic is more advanced (Clayton et al., 2024).

## Data Availability

The data underlying this article will be shared on reasonable request to the corresponding author.The study reported in this manuscript was developed under a trial that was registered in the database clinicaltrial.gov with the number NCT03615027.
